# Optimizing global COVID-19 vaccine allocation: An agent-based computational model of 148 countries

**DOI:** 10.1371/journal.pcbi.1010463

**Published:** 2022-09-06

**Authors:** Qingfeng Li, Yajing Huang

**Affiliations:** Department of International Health, Johns Hopkins Bloomberg School of Public Health, Baltimore, Maryland, United States of America; The Rockefeller Foundation, UNITED STATES

## Abstract

**Background:**

Based on the principles of equity and effectiveness, the World Health Organization and COVAX formulate vaccine allocation as a mathematical optimization problem. This study aims to solve the optimization problem using agent-based simulations.

**Methods:**

We built open-sourced agent-based models to simulate virus transition among a demographically representative sample of 198 million people in 148 countries using advanced computational services. *All countries continuing their current vaccine progress* is defined as the baseline scenario. Comparison scenarios include achieving minimum vaccination rates and allocating vaccines based on pandemic levels.

**Findings:**

The simulations are fitted using the pandemic data from 148 countries from January 2020 to June 2021. Under the baseline scenario, the world will add 24.36 million cases and 468,945 deaths during the projection period of three months. Inoculating at least 10%, 20%, and 26% of populations in all countries requires 1.12, 3.31, and 5.00 million additional vaccine doses every day, respectively. Achieving these benchmarks reduces new cases by 0.56, 2.74, and 3.32 million, respectively. If allocated by the current global distribution, 5.00 million additional vaccine doses will only avert 1.45 million new cases. If those 5.00 million vaccines are allocated based on projected cases in each country, the averted cases will increase more than six-fold to 9.20 million. Similar differences between allocation methods are observed in averted deaths.

**Conclusion:**

The global distribution of COVID-19 vaccines can be optimized to achieve better outcomes in terms of both equity and effectiveness. Alternative vaccine allocation methods may avert several times more cases and deaths than the current global distribution. With reasonable requirements on additional vaccines, COVAX could adopt alternative allocation strategies that reduce cross-country inequity and save more lives.

## Author summary

Since early 2020, the global COVID-19 pandemic has imposed enormous health and economic burden. While non-pharmaceutical interventions (e.g., partial or full lockdown; social distancing) have successfully mitigated the virus spreading in many countries, vaccination is key to helping people return to their normal lives. Highly effective vaccines have been developed, but the quantity will not be enough to vaccinate every people in the world soon. As a result, global vaccine allocation strategies lead to profound consequences in terms of impacts, coverage, and equity. COVAX formulated global vaccine allocation as a complex optimization problem. To solve the optimization problem, we developed agent-based models to experiment with various allocation strategies. Using advanced computational services, our models are able to capture individual and national heterogeneity, reproduce observed pandemic trajectories, and conduct forecasts under hypothetical allocation scenarios. Our results show the considerable advantage of alternative allocation strategies over current strategy that overwhelmingly favors high-income countries. Our open-source simulation tool is publicly available. The results can be updated in a semi-automatic manner. Upon minor modifications, our simulation tool can also be used to assist policymakers in exploring other vaccine allocation strategies and mitigation measures.

## Introduction

The global COVID-19 pandemic has caused tremendous health, economic, and social upheaval around the world. As of July 2021, more than 180 million cases and 4 million deaths have been recorded around the world.^1^ Vaccination is essential to prevent further morbidity and mortality. Highly effective vaccines were developed at an unprecedented pace and are rapidly being rolled out. More than three billion vaccine doses have been administered thus far [1]. However, the progress is uneven across countries. While certain high-income countries quickly approach herd immunity, vaccination rates remain minimal in many low- and middle-income countries (LMICs). According to data compiled by Johns Hopkins University, the global average vaccination rate is about 10%, but there is persistent and substantial inequity across countries. About 47% of administered doses were in high-income countries, many of which have inoculated about half of their population, including Israel (60%), United Kingdom (51%), and United States (48%). LMICs have received only 53% of vaccine doses despite accounting for 84% of the global population. Vaccination rate was still below 5% in 64 countries.

In addition to the apparent equity concerns, uneven vaccination progress also has substantial implications on effectiveness [2]. According to the World Health Organization (WHO), the groups most at risk of mortality are adults above 65 and adults with preexisting health conditions. Considering the distribution of those characteristics across countries, prioritizing a certain minimum percentage across countries would help cover those high-risk populations, regardless of their residence. Vaccinating low-risk groups in some countries while leaving high-risk groups unvaccinated in other countries reflects a missed opportunity to save lives. The inequity, called "scandalous" by WHO, needs to be addressed immediately. Due to the limited manufacturing capacity of COVID-19 vaccines, there will not be sufficient vaccines for every country until 2024[3]. Therefore, it is critical to optimize vaccination prioritization strategies while accounting for both equity and effectiveness.

To facilitate international vaccination efforts, Gavi, Coalition for Epidemic Preparedness Innovations, and WHO are co-leading the COVID-19 Vaccines Global Access (COVAX) Facility. It is a scheme that aims to raise funding and promote fair and equitable distribution of covid vaccines [4]. Based on the ethical principle of equal proportional share across countries, COVAX adopted a fair allocation mechanism operationalized through a two-phase plan. Given the ubiquity of the threat, the first phase accords priority to equity. All countries will receive sufficient vaccines to cover 20% of their populations. In phase 2, allocation will be based on risk assessments, including population threats and vulnerabilities.

COVAX formulates vaccine allocation as a complex optimization problem. They use a technique called linear programming to solve the problem and find the optimal allocation under various constraints of supply, demand, preference, and vaccine characteristics. The details of the solver algorithm are proprietary and, therefore, not available. COVAX only reveals that the solver is based on the simplex algorithm. The limited information suggests that the solver may not fully account for the complex dynamics of global vaccine allocation.

The global vaccination process involves a large number of national and international stakeholders. The process exhibits many complex characteristics, such as non-linearity, feedback loops, spontaneous order, adaptability, and self-organization. These features call for a systems science computational approach. For such complex issues, conducting experiments to identify the most effective of potential alternatives, especially accounting for the combined effects of all key factors, is preferable. Yet, the real world presents financial, technical, and ethical barriers that impede an experimental approach. Fortunately, this exact type of experiment can be done by creating a virtual environment, such as a computer-simulated model of the real world. In many other industries, alternative plans are simulated and compared before the best option is selected. For instance, simulators have long been a staple in planning space explorations in NASA [5]; US Department of Health and Human Services relies on simulation modeling to make effective recommendations on tobacco control [6]; the effects of new drugs are simulated in computational emulators before testing in human bodies [7].

There has been growing interest in using simulation tools to study pandemic models, particularly compartmental models, including the basic Susceptible-Infectious-Recovered (SIR) models and their variants. Agent-based modeling (ABM) and differential equation modeling (DEM) are two of the most frequently used approaches. With a long history in social science and epidemiology, DEM can encompass a wide range of feedback effects, but it is limited in handling heterogeneity within compartments [8]. It was computationally inexpensive, which facilitated its extensive application before advanced computational power became available to the general public; and many recent studies still used it to optimize public health interventions, such as influenza vaccination [9].

As a systems science-based computational model, ABM has been extensively used to study the COVID-19 pandemic and to simulate vaccination strategies, thanks to their capacity to simulate complex interactions between agents with heterogeneous characteristics and capture emergent phenomena [10,11]. Most existing models broadly fall into two categories: highly abstract models and local models. The former is frequently used to address hypothetical comparisons in abstract settings [11–13]. These models usually do not utilize real-world data, such as temporal and geographic distribution of population and cases. On the other hand, local models incorporate a diverse range of local contexts and real epidemiological data [14,15]. High-resolution and realistic representations of local areas offer opportunities to inform local health authorities [16]. These models focus on the pandemic and related factors in certain settings, such as schools and workplaces [17,18]. Despite advantages in closely capturing real-world communities and interaction patterns, local models undermine the strengths of systems science simulations. Local areas are closely integrated with surrounding areas, and separate models may miss certain key dynamics.

Therefore, the goal of the present study is to develop mathematical simulations to quantitatively compare and optimize vaccination strategies. Taking advantage of advanced computational services, we built one of the largest simulations on global COVID-19 transmission in 148 countries that account for over 90% of the global population and 95% of global COVID-19 cases. The models will be parameterized using real-world data and validated through cross-validations.

## Methods

To gain insights into global vaccine distribution, we need a global model based on real-world pandemic data. The model should also incorporate essential components of disease transmission, such as international travel. Such a global system of national and subnational models is computationally expensive to develop and implement. Fortunately, the increasing availability of advanced computational services has made the global modeling approach more feasible. The following paragraphs present major model components, their real-world counterparts, and data sources.

### Overall structure

**Fig 1:**
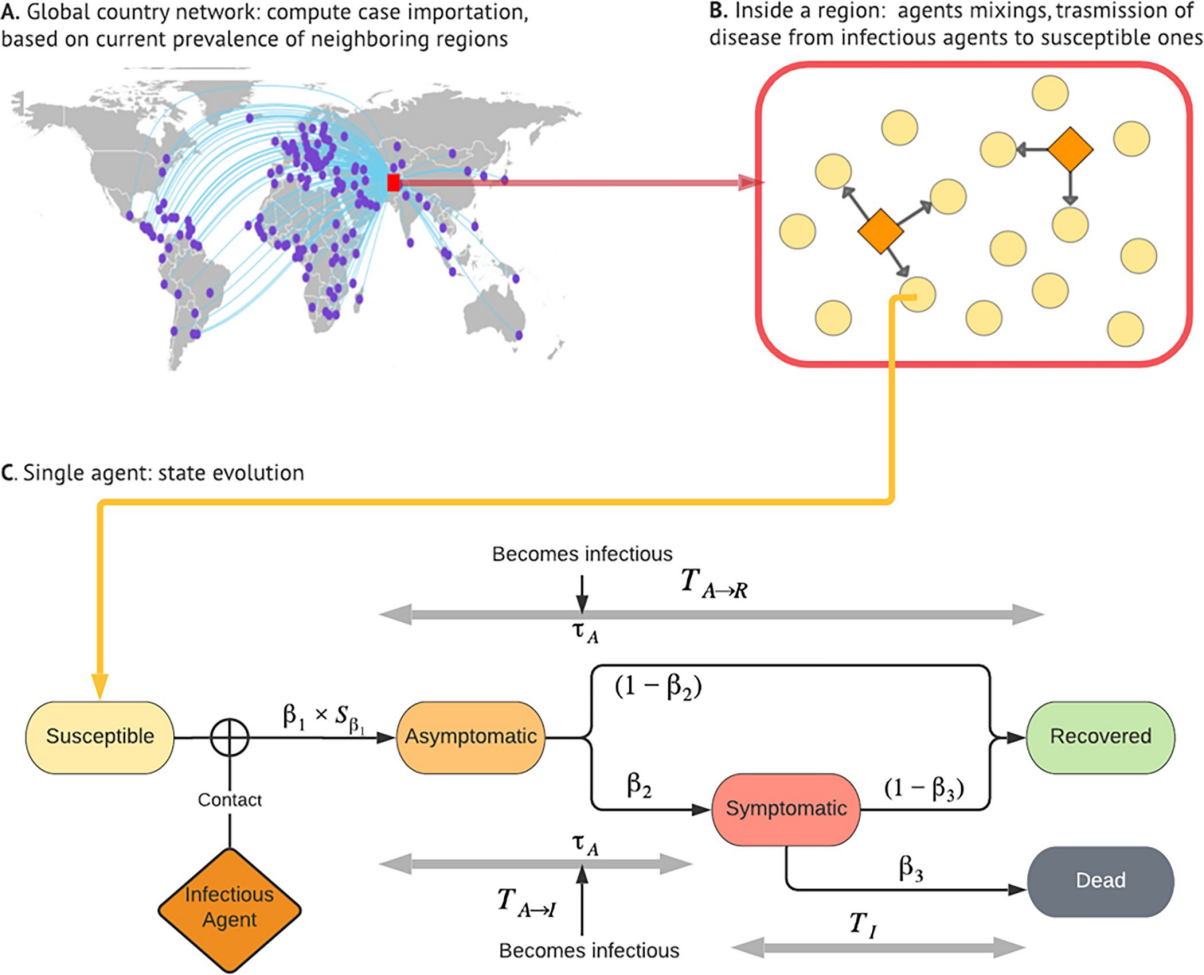
Simulation Framework. Note: the global map was made using the R package “maps”. The original source of the base layer is Natural Earth, whose Terms of Use explicitly state that "No permission is needed to use Natural Earth. Crediting the authors is unnecessary." https://www.naturalearthdata.com/about/terms-of-use/.

Our simulations account for the COVID-19 pandemic, associated factors, and their complex dynamics at the global, national, and individual levels (Fig 1) [19]. The first level consists of Susceptible-Exposed-Infectious-Removed (SEIR) models for 148 countries. One exception is the United States, where models are at the state level. However, the report still uses the term "national models" for simplicity. These separate national models are connected by international travel. Countries are selected for this study based on population size and COVID-19 prevalence. The 148 countries make up more than 90% of the global population and over 95% of COVID-19 cases in the world.

### Scale of simulations

A simulation’s scale refers to the agent size or the number of real-world individuals represented by an agent in the simulation. Choosing the appropriate scale is a major challenge for simulation studies. The synthetic generation of a one-to-one virtual population requires forbiddingly high computational demand. Using too few agents may exaggerate stochastic variability and generate biased findings, particularly for relatively rare events. Thanks to access to the enormous computational power provided by Maryland Advanced Research Computer Center, we were able to use 1 million agents per country (or per state for the US). This implies a reasonable scaling factor, defined as the number of real-world people represented by an agent in the simulation, for most countries. In total, our models simulate the virus transmission among 198 million people. To our knowledge, this is one of the largest simulation studies on COVID-19 transmission and vaccination.

### Agents

An ABM consists of dynamic agents. In this study, an agent represents a person that has an epidemiological status and demographic attributes. Potential epidemiological statuses include susceptible, infected but without symptoms, infected with symptoms, recovered, and dead. The study distinguishes between symptomatically and asymptomatically infected people due to the significant implications on virus transmission and control. Fig 1C illustrates the transition among different virus statuses.

Demographic attributes include sex, age group (children, adults, and seniors), and race. These three attributes have been associated with biological and behavioral factors that affect virus vulnerability. Race was only considered in the United States due to the lack of quality data in other countries. The COVID-19 pandemic data came from Johns Hopkins University [1]; demographic data were obtained from the World Population Prospects [20]. When sex- and age-disaggregated COVID-19 data were not available, we used the average disaggregation calculated from countries in the same WHO region.

### Virus transmission parameters

A diverse set of sources were used to obtain the ranges of parameter values. Major indicators of virus transmission include the transmission probability per contact, proportion of infections that eventually show symptoms, duration of asymptomatic period, time between infection and becoming infectious, and duration of symptomatic infection. The simulations also account for key behavioral indicators. Table 1 presents a list of those indicators, their definitions, and data sources. Sex and age have been identified as important risk factors for COVID-19 infection and mortality across countries. The relative risks in infection between males and females and between age groups are incorporated in the simulations. The notations for the transmission probability per contact was defined for senior females. Instead of defining parallel notations males and other age groups, a male-to-female ratio and several age-specific ratios (compared to seniors) were used to model the transmission probability for males and other age groups. Accordingly, in our simulations, we first vaccinate seniors and only move on to other age groups after vaccinating all seniors. This is consistent with the vaccination order in nearly all countries.

**Table 1:** Selected simulation model parameter values and sources.

Parameter (notation)	Description	Values or distribution	Reference
*β*_1_	Transmission probability per contact of senior females	[0.006,0.02]	Estimated from parameter fitting
$S_{\beta_{1}}$	Scale factor for *β*_1_, transmission probability relative to that of senior females (which means transmission probability per demo-group is $\beta_{1}\times S_{\beta_{1}}$)	[0,1]	Estimated from COVID sex and age disaggregated data of [26]
*β*_2_	Proportion of infected agents that eventually show symptoms	0.45	[27]
*T_A→I_* *T_A→R_*	Duration of asymptomatic period until symptoms start to show (if the next state is I), or until agents recover (if the next state is R)	If the next state is I, lognormal(1.63,0.5^2^); If the next state is R, lognormal(2.23,0.2^2^); Cut off at 21 days.	[28],[29]
*τ_A_*	The day when an asymptomatic case become infectious	If the next state is I, 2 days before turning into I, or day 1; If the next state is R, day 3.	[30],[31],[29]
*β*_3_	Proportion of symptomatic cases that result in death	[0,0.1]	[26]
*T_I_*	Duration of symptomatic infection	lognormal(2.04,0.4^2^); Cut off at 21 days.	[32]

Note: refer to the code and readme file on GitHub for a full list of parameters and their definitions and distributions.

### Vaccine efficacy

The study considers two types of vaccines, one with an efficacy of 65.8% (representing Johnson & Johnson) and the other with 95% efficacy (representing the mRNA-platform vaccines from Moderna and Pfizer-BioNTech) in preventing COVID infection [21–23]. Given our focus on vaccine distribution strategies, it is beyond the scope of the present study to explicitly account for vaccine heterogeneity in terms of effectiveness, platform, duration of protection, and geographic distribution. The vaccine data come from the Center for Government Excellence at Johns Hopkins University.

### Travel

For an international model consisting of individual national models, it is critical to account for cross-country travel. Due to a lack of detailed travel data, we focus on the main effect of traveling: bringing in new cases. The effect of travel can be quantified by the number of agents infected by imported cases, which is estimated by the weighted sum of COVID-19 prevalence in neighboring regions. The weights are computed as the inverse of flight hours between the capitals of regions, and they represent the effective proximity between regions.

The overall impact on the development of the epidemic depends on the ratio of imported cases to domestic cases. For countries in an ongoing outbreak, the impacts of imported cases can be minimal. However, imported cases may be a more significant issue if a country has nearly eliminated the virus. Global travel data for 2021 were extracted using an application programming interface (API) provided by kiwi.com.

### Simulation scenarios

*All countries continuing their current vaccination progress* is defined as the baseline scenario. This study simulated two major groups of scenarios, prioritizing equity and effectiveness respectively.

The first group of scenarios allocates vaccines proportional to population, which is similar to COVAX’s phase 1 plan. Since COVAX has not published the details of its algorithm, we set a series of numerical goals. WHO called for inoculating at least 10% in all countries by September 30, 2021. This target forms the first scenario in our simulation. The second scenario doubles the target to 20%. The third scenario of 26% is also included since it implies the same vaccine demand as scenarios in the second group discussed below. The simulations use observed data up to June 30, 2021. This leaves three months to achieve the September minimum inoculation target set by WHO. Accordingly, all our simulations are for a three-month projection period.

The second group of simulations focuses on effectiveness, which is similar to COVAX’s phase 2 plan. COVAX broadly proposed potential parameters to assess threat and vulnerability (e.g., effective reproductive number, health system functions). The implementation details will not be finalized until closer to the end of phase 1. In this study, we use cases and case prevalence to proxy risk assessment. In particular, five million additional vaccines (the same as the 26% scenario above) are distributed on a daily basis to countries per four metrics: current biweekly number of new cases, current biweekly case prevalence, projected number of new cases, and projected case prevalence. In sum, the differences between those simulation scenarios are in the vaccine allocation methods during the project period.

### Simulation procedure

The simulation is divided into several steps. We first use the root mean square error (RMSE)-minimizing approach to find the parameter values that give the best fit for pandemic curves observed between 1/1/2020 and 6/30/2021. This stage is further divided into two periods. In the first period, the simulation was performed on disconnected regional models with the same default demographic characteristics. It is for setting the model for parameters that are significant at governing the shape and size of the outbreak curve. We used a grid search with 6300 combinations of parameters to find values that provide both in- and out-of-sample agreements with observed pandemic trajectories for each country. Each combination was repeated with 10 different random seeds, implying a final grid search among 63000 simulations for each region.

In the second period, traveling, vaccination and regional demographic characteristics are added to the models, and to account for changes in the modelled curves that are introduced, parameters are slightly adjusted to ensure a good fit to the data curves. This fitting stage started when the vaccination rate reached 3% in the region or 5/31/2021, whichever came first.

The subsequent 40 days are used for out-of-sample validation. The last step is the simulation of the vaccine allocation scenarios discussed above. Pandemic metrics are monitored to evaluate the impacts of additional vaccines.

Like in most large-scale simulation studies, the complexity of the model setting forbade us from mathematically quantifying the uncertainty in the results. As a sensitivity analysis, we re-ran the model 20 times using different random seeds. The choice of seeding did not pose a significant impact on our results since the results for each scenario varied only marginally across iterations. More importantly, the difference (in absolute and relative terms) between different scenarios remained nearly constant. That confirmed the robustness of our models, parameterization, and findings.

The models were developed using Python, and the simulations were carried out on a cluster provided by the Maryland Advanced Research Computer Center. The final results were obtained using a cluster of 24 cores and 117 GB RAM. The computation time for a three-month projection is approximately 4 hours.

### Patient and public involvement

Patients or the public were not involved in the design, or conduct, or reporting, or dissemination plans of our research.

### Role of the funding source

The funders of the study had no role in study design, data collection, model development, result interpretation, or report writing.

## Results

**Fig 2:**
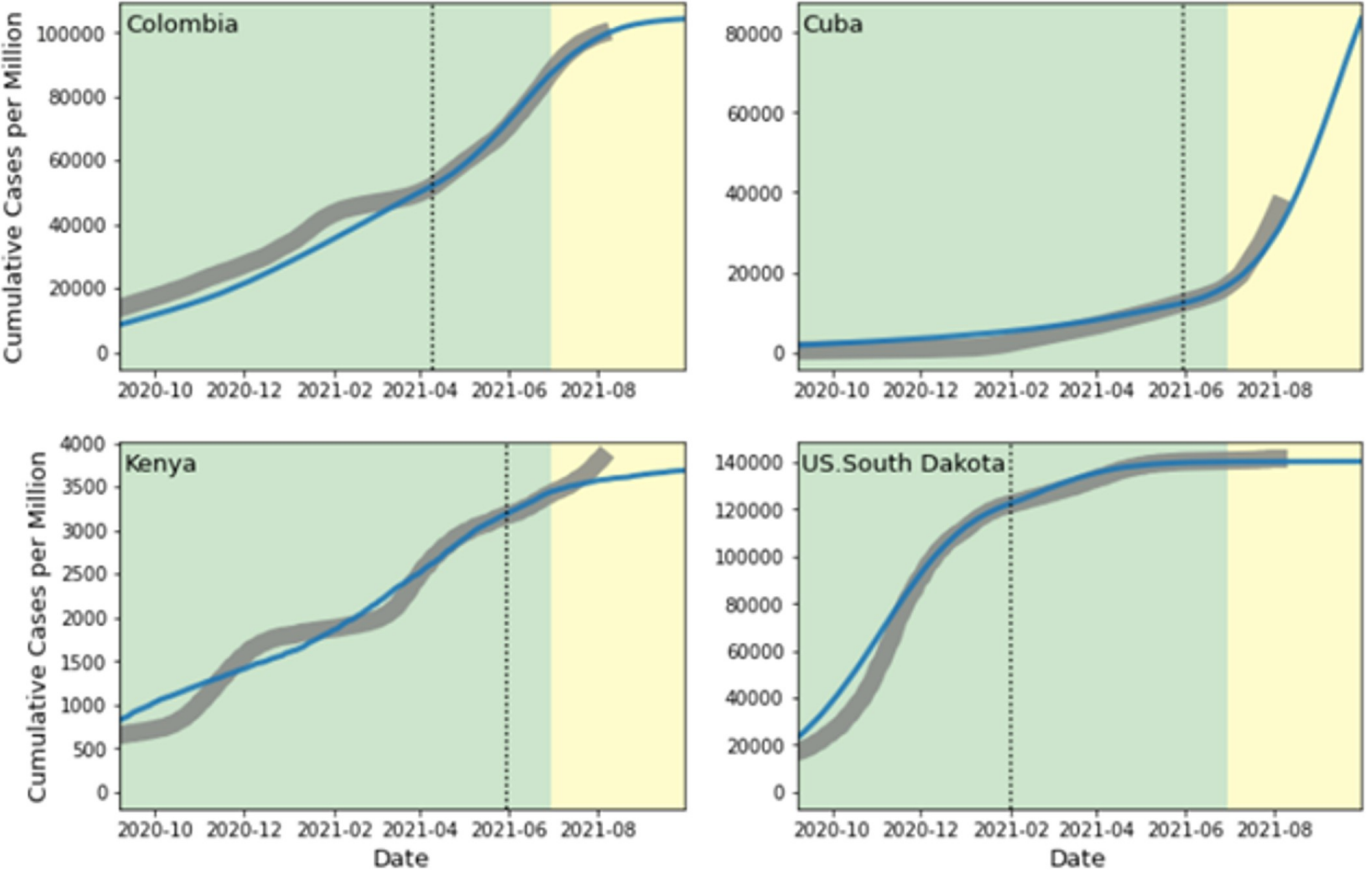
Model fitting for selected countries. Model fitting (shaded green) and out-of-sample validation (shaded yellow) in four selected countries. The grey curves are the observed cumulative cases per million, and the blue curves are the simulated results. The dotted lines signal the days when traveling, vaccination, and regional demographic characteristics are added to the simulations.

The simulation settings closely fit the observed pandemic data. Fig 2 illustrates the model fitting and out-of-sample validation for four selected countries. The regions were selected to represent two major patterns of agreement between observed data and simulated trajectories. The simulation performs well in most regions throughout the study period. Three plots in Fig 2 (Columbia, Cuba, and South Dakota, US) illustrate a consistent agreement observed in over 85% of regions included in the study. The plot from Kenya represents a small number of countries where the agreement appears to be weak. The model struggles to handle the multi-wave nature of the COVID-19 trajectory in the country. The fit may be improved by adding time variability to the transmission rate. However, the resulting complexity may lead to unintended consequences for other countries. Customizing simulation settings for individual countries may improve the fitting, but we left it to future research, given the overall satisfying accuracy that we have achieved with the current settings.

**Fig 3:**
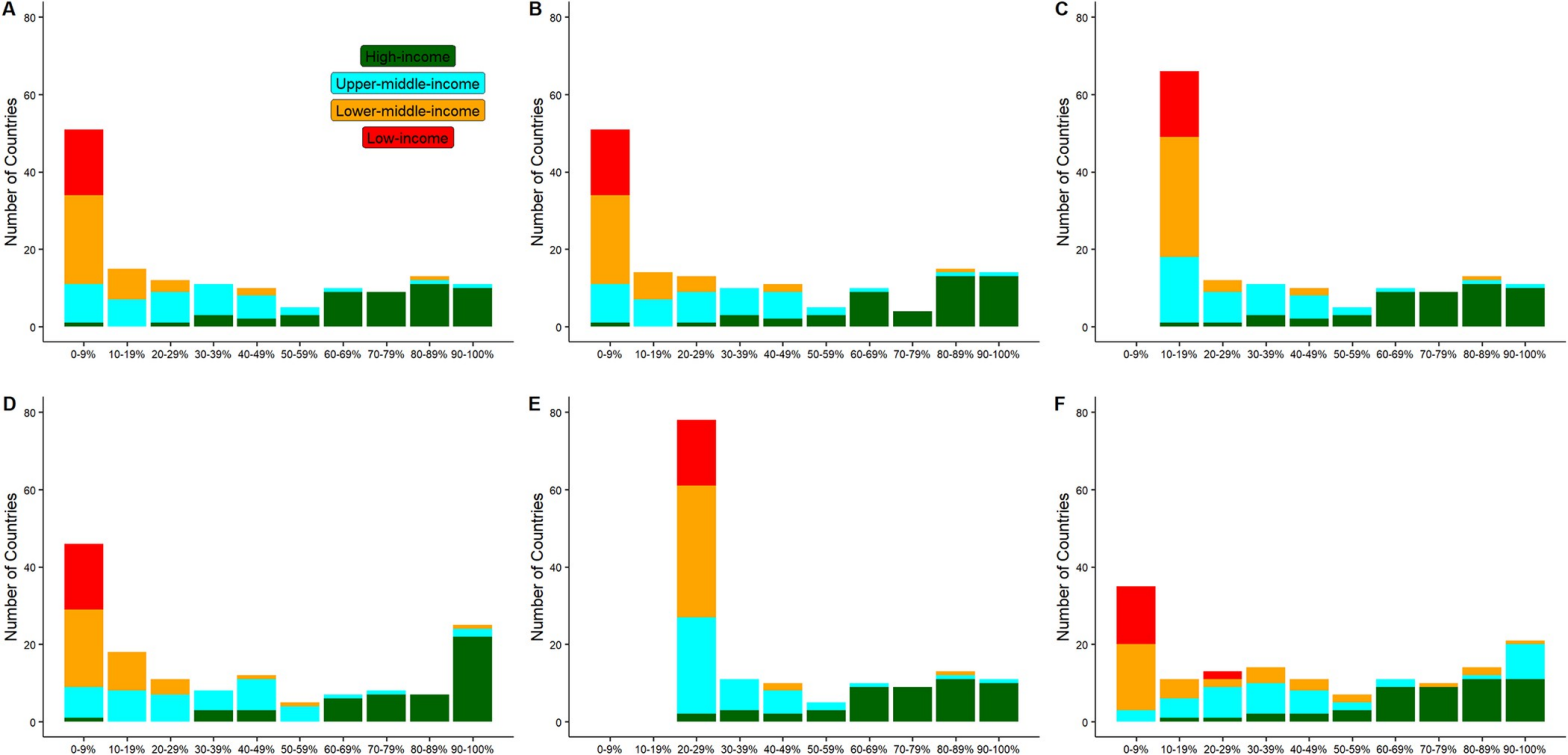
Number of countries by vaccination rate by the end of the projection period. (A) baseline. All countries continue their current vaccination progress for the projection period of three months. No additional vaccines. (B) 1.11 million additional vaccines every day are distributed per current global distribution. (C) 1.11 million additional vaccines every day are distributed to achieve a minimum vaccination rate of 10% for all countries. (D) 5.00 million additional vaccines every day are distributed per current global distribution. (E) 5.00 million additional vaccines every day are distributed to achieve a minimum vaccination rate of 26% for all countries. (F) 5.00 million additional vaccines every day are distributed per national projected cases.

The baseline scenario projects 24.36 million cases and 468,945 deaths for the three-month projection period (Table 2). About 53% of the cases occur in five countries (India, South Africa, Iran, Indonesia, and Malaysia). An even higher percentage (57%) of deaths come from the five countries most affected by COVID-19 (South Africa, India, Iran, Indonesia, and Brazil). Following their recent vaccination progress, many LMICs will keep falling behind (Fig 3). Nearly all low-income countries (LICs) are projected to fail to reach 10% vaccination coverage. The disparity between income levels will remain.

**Table 2:** Cases and deaths during the projection period in simulated scenarios.

Scenario	Total Vaccines	Additional Vaccines	Cases		Averted cases			Averted cases (%)			Deaths		Averted Deaths			Averted Deaths (%)		
			CAM	AAM	CAM vs baseline	AAM vs CAM	AAM vs baseline	CAM vs baseline	AAM vs CAM	AAM vs baseline	CAM	AAM	CAM vs baseline	AAM vs CAM	AAM vs baseline	CAM vs baseline	AAM vs CAM	AAM vs baseline
Baseline	18,678,746		24,363,731								468,945							
Minimal 10% vaccination	19,794,450	1,115,704	23,898,862	23,339,854	464,869	559,008	1,023,877	1.9	2.3	4.2	458,208	444,766	10,737	13,443	24,180	2.3	2.9	5.2
Minimal 20% vaccination	21,992,586	3,313,840	23,692,424	20,949,427	671,307	2,742,997	3,414,304	2.8	11.3	14.0	452,256	399,597	16,690	52,659	69,348	3.6	11.2	14.8
Minimal 26% vaccination	23,678,994	5,000,248	22,911,520	19,590,241	1,452,211	3,321,279	4,773,490	6.0	13.6	19.6	443,314	367,591	25,632	75,723	101,354	5.5	16.1	21.6
Allocation per recent cases	23,678,994	5,000,248	22,911,520	17,773,307	1,452,211	5,138,213	6,590,424	6.0	21.1	27.1	443,314	322,316	25,632	120,998	146,629	5.5	25.8	31.3
Allocation per recent prevalence	23,678,994	5,000,248	22,911,520	17,749,574	1,452,211	5,161,947	6,614,158	6.0	21.2	27.1	443,314	323,609	25,632	119,705	145,337	5.5	25.5	31.0
Allocation per projected cases	23,678,994	5,000,248	22,911,520	15,159,537	1,452,211	7,751,983	9,204,194	6.0	31.8	37.8	443,314	277,856	25,632	165,458	191,089	5.5	35.3	40.7
Allocation per projected prevalence	23,678,994	5,000,248	22,911,520	16,106,303	1,452,211	6,805,218	8,257,429	6.0	27.9	33.9	443,314	298,914	25,632	144,400	170,032	5.5	30.8	36.3

CAM: current allocation method; AAM: alternative allocation method

The following scenarios address the disparity by setting a minimum vaccination percentage for all countries. In the first scenario, which aims to inoculate at least 10% of every country by the end of the project period, 1.11 million additional vaccine doses are required every day. Achieving this goal will avert new cases and deaths by 1.02 million and 24,180, respectively. The reductions mainly occur in currently under-vaccinated countries. The countries benefiting most are South Africa, Indonesia, Cuba, Bangladesh, and Mozambique. As a comparison, if allocated per the current global distribution, the same amount of vaccines will only avert 0.46 million cases and 10,737 deaths.

Moreover, the relative reduction in deaths (4.6%) is twice the reduction in cases (2.3%). This is because the simulations prioritize vaccinating the elderly, who have a higher case-fatality ratio than the general population. Another reason is that beneficiary countries tend to have a higher case-fatality ratio than other countries. The results demonstrate the benefits of WHO’s call for global minimum coverage of vaccination. It promotes equity by eliminating countries with less than 10% vaccination. It also improves effectiveness by allocating vaccines to countries and populations where vaccines have larger chances to prevent infections and deaths. As a result, for the same vaccine requirement, the averted cases and deaths would more than double.

Increasing the minimum target to 20% corresponds to 3.41 million averted cases and 69,348 averted deaths as compared to the baseline. The requirement for additional daily vaccines more than doubles to 3.31 million. Allocating that amount of vaccines per the current global distribution will only avert 0.67 million cases and 16,690 deaths.

A more ambitious target of 26% requires 5.00 million vaccine doses every day. Achieving the target is projected to avert 4.77 million cases and 101,354 deaths. If the same amount of vaccines are allocated per the current global distribution, the averted cases and deaths will dramatically reduce to 1.45 million and 25,632, respectively.

Subsequent effectiveness-oriented scenarios explore other approaches to allocating the extra 5 million vaccines each day. If the allocation is based on the number of cases in the recent month, 6.59 million cases and 146,629 deaths will be averted, which is a substantial increase from the scenarios discussed above. Top beneficiary countries include South Africa, Iran, Cuba, Malaysia, and Indonesia, Bangladesh, and Brazil.

Among the simulated scenarios, the most cases and deaths are averted if the allocation is based on projected cases for the three months. In that scenario, the averted cases reach 9.20 million, which is 6.33 times higher than allocating those vaccines per the current global distribution. The ratio is even higher at 7.46 for averted deaths. Top beneficiary countries are still among the currently under-vaccinated countries. Allocation per projected prevalence essentially prioritizes high-risk nations, which is expected to maximize the averted cases and deaths. This is not the case in our simulations because our allocation is determined at the beginning of the projection period. It can be inferred that the maximum number of averted cases and deaths will be achieved if the allocation is dynamically adjusted (e.g., on a daily basis) per the projected prevalence.

The comparison between the 26%-minimum and projected-cases scenarios also reveals the tradeoff between equity and effectiveness. The former allocation method guarantees a minimum vaccination of 26% across all countries (Fig 3C.) While the latter scenario averts more cases (9.20 million vs. 4.77 million) and saves more lives (191,089 vs. 101,354), it contributes much less to reducing inequity across income levels. The vaccination rate remains below 20%, or even 10%, in many low- and middle-income countries in that scenario (Fig 3).

The simulated scenarios overperform the baseline scenario and current global allocation in terms of both equity and effectiveness. While it is important to increase vaccine manufacturing capacity, optimal allocations have the potential to avert substantially more cases and deaths for a given vaccine supply.

## Discussion

As one of the first global simulations on COVID-19 vaccine allocation, the study compares various vaccination strategies based on real-world data on travels, epidemiology, and demography. We utilize advanced computational services to conduct one of the largest computational simulations on the COVID-19 pandemic.

Due to the tremendous implications, both policymakers and researchers have shown intense interest in COVAX strategies. Many journal articles and news reports have been published on the principles of COVAX strategies as well as potential improvements. However, quantitative assessment is still limited. To our knowledge, our study is among the first global simulations that numerically compare vaccine allocation strategies that are in line with the COVAX principles.

As a simple approach to promoting equity, ensuring a minimum vaccination coverage for all countries also has effectiveness advantages. This allocation approach prioritizes vaccination in countries with minimal vaccine coverage, where vaccines are more likely to be administrated to high-risk individuals. For a daily allocation of only 1.12 million extra vaccines, about 1.02 million cases and 24,180 lives would be averted. Furthermore, the benefits mostly go to severely under-vaccinated countries and populations. The allocation of 5 million doses every day per pandemic level has the potential to prevent 9.20 million cases and save 191,089 lives.

Beyond the substantive insights from the simulations, this study also has methodological contributions. Our results suggest that it is possible to closely fit the pandemic data using expanded SEIR models. A reasonably large population in the simulation (i.e., a small scaling factor) allows researchers to create a virtual world with sufficient granularity. Methodologically, the study contributed a simulation tool that has been thoroughly validated using real-world pandemic, demographic, and travel data around the world. Straightforward adaptations to the tool would assist policymakers and researchers in exploring a wide range of virus-controlling options. For example, in order to incorporate the impacts from the more contagious variants, such as the Delta and Omicron variants, a user only needs to enter new transmission parameters and vaccine effectiveness while keeping most of the model structure and setting intact [24]. Then, the code shared by the authors should be able to re-calibrate the model and update the results.

Although extensively used by researchers and policymakers, there is no open-source code written in a general programming language to simulate the SIR model or its variants (except toy models). Our code will hopefully inspire and facilitate further development and more applications of SIR models. We recommend two directions for future research: making the ABM models accessible to a broader group of potential users who may not have access to advanced computational services; adapt the models to simulate national-level COVID control strategies. Our models capture individual heterogeneity, different network topologies, complex interactions, and global connections. But that came at the cost of demanding computational requirements. The dependence on advanced computational services makes the models less accessible to potential users, such as policymakers and researchers. Future research may try to further improve the computational efficiency of the models. A potential solution is to divide the computations into pre-and post-processing. This may result in some loss of accuracy, with the benefit of dramatically increased computational efficiency. This approach is widely used in Bayesian models and machine learning when the full models are too computationally intensive to be practically useful [25].

The second direction involved adapting the ABM models to incorporate national-level heterogeneity. More than two years into the COVID pandemic, countries around the world have explored various approaches to bring the virus under control or even eliminate it completely. There are many statistical analyses based on observed data to evaluate the effectiveness of those interventions. It is possible to adapt the models and calibrate them using the observed data. Such global models with nationally calibrated settings and parameterizations could provide insights into the effectiveness of those interventions.

Despite our extensive effort to diagnose and improve the models, the study is not without limitations. First, like all simulations, this study only captures the essential components of the systems. Thus, potentially important factors that may affect the outcomes may have been left out. For example, there is substantial heterogeneity in vaccine effectiveness and national regulatory requirements. The models are built for one-dose vaccines (or mathematically equivalently, for the second dose of two-dose vaccines). Second, for a global analysis, it is beyond the scope of the study to evaluate the data quality from every country and make adjustments accordingly. As a result, the findings may suffer from biases if pandemic data are underreported or inaccurate. The same goes for travel data. Our models focus on flight travel between countries and only include land travels between states in the US. While acknowledging the importance of other modes of transportation, particularly between neighboring countries, we did not find a global-level standardized database for those travels. Assembling such information from various sources and checking their quality is beyond the scope of the current study. Third, the current model setting may not sufficiently account for multiple waves, particularly when the scale (e.g., peak and duration) substantially varies by wave. In these cases, our model is more affected by the most recent or current wave than previous waves. Lastly, our model does not account for different virus variants, mainly due to the lack of reliable data at the national and international levels.

The success of COVID-19 vaccine development resulted from the joint effort by scientific researchers, pharmaceutical companies, and political support. The same synthesis is needed to deliver vaccines to every person around the world. While increasing manufacturing capacity to produce vaccines for all populations is the long-term goal, optimizing vaccine distribution is critical to stopping the pandemic and reducing inequity in the near term.

## Supporting information

S1 Appendix 148 countries included in the study. (DOCX)
