# Supplementary material for: Optimizing global COVID-19 vaccine allocation: An agent-based computational model of 148 countries
Source: PLoS Comput Biol. 2022 Sep 6;18(9):e1010463. doi: 10.1371/journal.pcbi.1010463 (PMC9447912; doi:10.1371/journal.pcbi.1010463)
Supplement: S1 Appendix — (DOCX) [file pcbi.1010463.s001.docx]

**Appendix: 148 countries included in the study**

Colombia

Comoros

Congo (Brazzaville)

Costa Rica

Cote d'Ivoire

Croatia

Cuba

Cyprus

Czechia

Denmark

Djibouti

Dominican Republic

Ecuador

Egypt

El Salvador

Equatorial Guinea

Estonia

Ethiopia

Finland

France

Gabon

Gambia

Georgia

Germany

Ghana

Greece

Guatemala

Guinea

Guinea-Bissau

Guyana

Haiti

Honduras

Hungary

Iceland

India

Indonesia

Iran

Iraq

Ireland

Israel

Italy

Jamaica

Japan

Jordan

Kazakhstan

Kenya

Korea, South

Kosovo

Kuwait

Kyrgyzstan

Latvia

Lebanon

Libya

Lithuania

Luxembourg

Madagascar

Malawi

Malaysia

Mali

Malta

Mauritania

Mexico

Moldova

Monaco

Mongolia

Montenegro

Morocco

Mozambique

Namibia

Nepal

Netherlands

Nicaragua

Nigeria

North Macedonia

Norway

Oman

Pakistan

Panama

Papua New Guinea

Paraguay

Peru

Philippines

Poland

Portugal

Qatar

Romania

Russia

Rwanda

Saint Lucia

Sao Tome and Principe

Saudi Arabia

Senegal

Serbia

Singapore

Slovakia

Slovenia

Somalia

South Africa

South Sudan

Spain

Sri Lanka

Sudan

Suriname

Sweden

Switzerland

Syria

Thailand

Togo

Trinidad and Tobago

Tunisia

Turkey

Uganda

Ukraine

United Arab Emirates

United Kingdom

United States

Uruguay

Uzbekistan

Venezuela

Zambia

Zimbabwe
